# The Physician’s Hand-book of Practice, for 1860

**Published:** 1860-01

**Authors:** 


ART. VIII.—*The Physician's Hand-book of Practice, for* 1860. By WILLIAM ELMER, M.D., and LEWIS ELSBERG, M.D., pp. 190. New York, 1860.

OF all the little pocket annuals that have met our eye this is, undoubtedly, the most complete, although, from its size, perhaps not the most convenient. To the young practitioner it cannot prove otherwise than extremely valuable. The scope of the book is best indicated by the nature of its contents, the most important of which are: A calendar, for 1860; classification of diseases; eruptive, and other fevers; diseases of the skin, vessels, and internal organs; Hall's ready method; medicinal weights and measures; abbreviations of the medical properties of remedies; list of remedial agents; list of incompatibles; medicated baths; art of writing prescriptions; poisons and their antidotes; diagnostic examination of the urine; explanation of signs; names; addresses; bills and accounts; record of practice and treatment; obstetric calendar; obstetric record; wants, and general memoranda; nurses; and blanks. We wish this hand-book an extensive circulation.

